# Revaluating the survival effects of International Federation of Gynecology and Obstetrics 1988 stage IIIA criteria for endometrial cancer

**DOI:** 10.4274/jtgga.2017.0033

**Published:** 2017-09-01

**Authors:** Osman Türkmen, Alper Karalok, Derman Başaran, Günsu Kimyon, Gizem Kul, Gökhan Tulunay, Işın Üreyen, Taner Turan

**Affiliations:** 1 Department of Gynecologic Oncology, Etlik Zübeyde Hanım Women’s Health Training and Research Hospital, Ankara, Turkey

**Keywords:** Endometrial cancer, peritoneal cytology, surgical staging

## Abstract

**Objective:**

This study aimed to define factors that affected survival in the International Federation of Gynecology and Obstetrics (FIGO) 1988 stage IIIA endometrial cancer (EC).

**Material and Methods:**

The study included patients with EC who underwent surgery between 1992 and 2013. Patients with adnexal metastases, uterine serosal involvement or positive peritoneal cytology (stage IIIA disease according to the former 1988 FIGO staging system) were selected for further analysis. Clinical and pathologic factors associated with progression-free survival (PFS) were evaluated using univariate and multivariate statistical tests.

**Results:**

Seventy-seven patients with stage IIIA disease according to the 1988 FIGO staging system were included. The median follow-up was 37 months (range, 1-175 months) and recurrence was detected in 19 patients. Univariate analysis revealed that the presence of uterine serosal invasion and advanced histologic grade (grade 1-2 vs. grade 3) were associated with diminished PFS (p=0.001, p=0.047). The presence of adnexal involvement and positive peritoneal cytology had no statistically significant influence on PFS (p=0.643 and p=0.795, respectively).

**Conclusion:**

In patients with stage IIIA EC according to the FIGO 1988 staging system, only uterine serosal involvement was related with adverse oncologic outcomes, not adnexal involvement or presence of positive cytology.

## INTRODUCTION

Endometrial cancer (EC) is the most frequent gynecologic tumor and the fourth most common malignancy in women (1). EC is mostly seen in the sixth and seventh decades and 95% of patients are aged over 40 years (2). According to International Federation of Gynecology and Obstetrics (FIGO), 83% of patients are diagnosed at stage I and II (3). Endometrioid-type constitutes 75% of the histologic types. Conversely, non-endometrioid types are high-grade aggressive tumors and mostly diagnosed at advanced stages (4, 5).

After 1988, in accordance with FIGO’s proposition, EC has been staged surgically. The surgical stage is the most significant factor that determines prognosis (6). According to the staging system developed in 1988, peritoneal cytology was considered as an important variable, and positive peritoneal cytology, adnexal metastasis, and serosal invasion were defined as stage IIIA (7). Various studies showed that peritoneal cytology has important implications for prognosis (7, 8, 9). On the contrary, other investigators showed that positive cytology does not determine survival in the absence of serosal invasion and adnexal metastasis (10, 11, 12). After several studies on the prognostic value of peritoneal cytology, FIGO revised the EC staging system in 2009 and peritoneal cytology was excluded from the staging criteria (13).

In this study, we purposed to evaluate the prognostic value of peritoneal cytology in 1988 FIGO stage IIIA for endometrial malignancy and to determine factors that affect survival in this stage.

## MATERIAL AND METHODS

This study included patients with EC who underwent surgery in our unit between 1992 and 2013. Patients with ovarian and tubal metastases, uterine serosal involvement or peritoneal cytology (stage IIIA disease according to the former 1988 FIGO staging system) were selected for further analysis. Patients with sarcoma or synchronized gynecologic tumors were excluded from the study. Patients with FIGO 1988 stage IIIA EC were re-staged according to FIGO 2009 criteria. Demographics, intraoperative findings, and surgico-pathologic results were collected from patient files, pathology results, and electronic database of the department of gynecological oncology.

All patients underwent hysterectomy and bilateral salpingo-oophorectomy and peritoneal cytologic sampling. Systematic lymphadenectomy was performed for patients in whom frozen section revealed a non-endometrioid adenocarcinoma, stage III disease, deep myometrial invasion, cervical involvement, tumor size >2 cm or extrauterine spread. We defined recurrence between the levator muscle and linea terminalis as pelvic recurrence, recurrence between the linea terminalis and diaphragm muscle as upper-abdominal recurrence, and all other recurrences as extra-abdominal recurrence. Ascites and peritonitis carcinomatosa were considered as upper abdominal recurrence. Liver parenchyma, skin and bone recurrence were considered as extra-abdominal recurrence. Progression-free survival (PFS) was defined as the time between primary treatment and recurrence or last visit. The time until death of disease or until the last visit was defined as disease-specific survival (DSS).

SPSS (SPSS Inc, Chicago IL, USA) version 15.0 was used for statistical analyses. Kaplan-Meier analysis was used for the computation of PFS and DSS. Survival curves were checked using the log-rank test. Prognostic factors were analyzed using the Cox regression model. Factors with a p value below 0.25 in the univariate analysis were included in the multivariate analysis. The cut-off for statistical significance was set at p<0.05. As this work represents a retrospective chart review, local ethics committee permission was not sought. However, all patients signed informed consent thereby allowing our institution to use their clinical data.

## RESULTS

Seventy-seven patients with stage IIIA EC according to the 1988 FIGO staging system were available for analyses. Data of 1413 patients who had at least extrafascial hysterectomy + bilateral salpingo-oophorectomy were obtained from the gynecological oncology clinic electronic database and patient’s files, retrospectively. Among these 1413 patients, 77 (5.4%) were at stage IIIA according to FIGO 1988 criteria. The median age was 59 years (range, 29-81 years). The pathologic characteristics of the patients are represented in Table 1. Sixty-six (85.7%) patients had endometrioid histology and more than half had deep myometrial invasion and serosal invasion. The mean tumor size was 36.4 mm (range, 16-60 mm).

Table 2 shows details of the pathologic features of the patients that were used to assign stage IIIA according to FIGO 1988. Isolated positive peritoneal cytology without serosal and adnexal involvement was positive in 11 (14.3%) patients. Isolated serosal invasion and isolated adnexal involvement were the cause of stage IIIA disease in 10 (13%) and 40 (51.9%) patients, respectively. Eleven patients who had isolated positive peritoneal cytology were re-evaluated using FIGO 2009 staging criteria and 6 patients were found to have stage IA disease, 4 patients were defined as stage IB, and 1 was evaluated as having stage II disease. Sixty-four patients underwent systematic lymph node dissection. Lymphadenectomy could not be performed in 13 patients due to medical co-morbidities. The median number of harvested nodes was 58 (range, 11-103). There was no significant association between positivity of peritoneal cytology and surgico-pathologic factors such as adnexal metastasis, serosal involvement, tumor grade, cervical involvement, and depth of myometrial invasion.

The decision of adjuvant treatment and type of adjuvant therapy was given in gynecologic oncology council according to the risk factors of patients, including grade, myometrial invasion, serosal involvement, and cervical involvement. Seventy-one patients received adjuvant treatment. Of these, 14 patients received chemotherapy, 50 were irradiated, and 7 received combination chemo-radiotherapy. Sixty-one (97.2%) patients had complete clinical response after adjuvant treatment, and 2 (2.8%) had progressive disease under treatment. After the disease progressed, these 2 patients refused further treatment and were lost to follow-up.

The median follow-up was 37 months (range, 1-175 months) and recurrence was detected in 19 (24.7%) patients. Five patients had extra-abdominal recurrence, 4 had pelvic, 4 had upper abdominal, 4 had both pelvic and upper abdominal, and 2 had both pelvic and extra-abdominal recurrence. The estimated 5-year PFS was 68.6%. There were no deaths due to disease during follow-up; therefore, factors that determined DSS were not studied in this paper.

**Figure 1:**
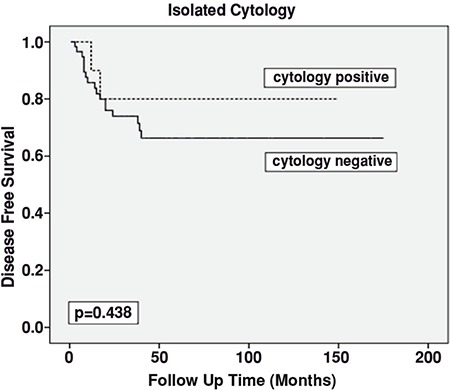
Survival effect of peritoneal cytology

Univariate analysis revealed that the presence of uterine serosal invasion and advanced histologic grade (grade 1 and 2 vs. grade 3) were associated with diminished PFS (p=0.001, p=0.047, respectively) (Table 3). The presence of adnexal involvement and positive peritoneal cytology had no statistically significant influence on PFS (p=0.643, p=0.795, respectively). Subgroup analysis showed that there was no statistically significant difference between patients with negative cytology and those with isolated positive cytology in terms of PFS (Figure 1). The 5-year PFS was 68.3% and 69.1% in patients with negative and isolated positive cytology, respectively. Age, tumor histology, depth of myometrial invasion (patients with uterine serosal invasion were excluded), cervical involvement, lymphovascular space invasion, and type of adjuvant treatment were not related with PFS (Table 3).

**Figure 2:**
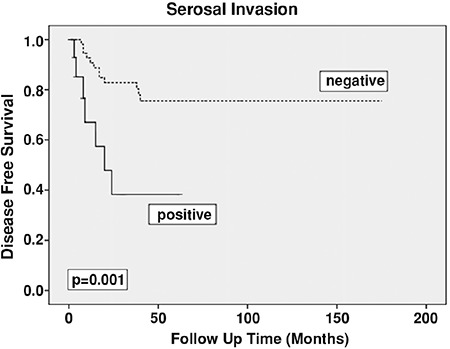
Survival effect of serosal invasion

Uterine serosal involvement, grade, cervical stromal invasion, and tumor type were used for the multivariate analysis model. Uterine serosal involvement was determined as the only independent adverse prognostic factor for recurrence (hazard ratio: 5.015, 95% confidence interval: [1.850-13.592]; p=0.002) (Table 4, Figure 2).

## DISCUSSION

Despite many studies on the subject, the prognostic value of peritoneal cytology in EC has not yet been proven. There are studies that describe the prognostic value of peritoneal cytology in EC (7, 8, 9). In addition, previous studies demonstrated that peritoneal cytology is an independent prognostic factor (14, 15, 16, 17). Garg et al. (8) defined peritoneal cytology as an independent prognostic factor in patients with EC limited to the uterus. The study contained 14,704 patients with tumors of variable histology that were limited to the uterus and revealed that peritoneal cytology was associated with diminished survival (8). In a study of 196 patients who had stage IIIA EC according to FIGO 2009 criteria, Milgrom et al. (18) showed that positive peritoneal cytology was related with cervical stromal invasion, ovarian and tubal involvement, and non-endometrioid type tumors. In our study, cervical invasion, adnexal involvement, serosal invasion, depth of myometrial invasion, and FIGO stage did not seem to have an effect on peritoneal cytology. This difference is related with the fact that we defined our study according to FIGO 1988 criteria, whereas Milgrom et al. (18) used FIGO 2009 criteria. In our study, there were only 11 patients with stage IIIA disease according to FIGO 1988 criteria because of isolated positive peritoneal cytology.

In our study, the 5-year PFS was 68%. Uterine serosal invasion was the only independent prognostic factor associated with PFS, whereas peritoneal positive cytology, adnexal metastasis, age, tumor histology, myometrial invasion depth, and lymphovascular space invasion were not associated with PFS. Milgrom et al. (18) showed that the 5-year PFS for stage IIIA patients according to 2009 criteria was 63%. In their study, the 5-year PFS was found as 39% in patients with positive peritoneal cytology and 69% for patients with negative peritoneal cytology (p=0.001). In another study, Garg et al. (8) reported that deep myometrial invasion, aggressive tumor type, absence of pelvic lymphadenectomy, and not receiving adjuvant radiotherapy were poor prognostic factors regarding PFS (p=0.014, p<0.001, p=0.005, p<0.001, respectively). They found that patients with positive peritoneal cytology had higher recurrence in the paraaortic lymph node region and peritoneal surface than those with negative peritoneal cytology; however, there was no difference regarding pelvic recurrence rates between these two patient groups. They also determined no meaningful higher recurrence rate in lymph node regions in patients with positive peritoneal cytology (p=0.438) (19). In our study, we found no retroperitoneal recurrence in our cohort. The absence of lymphatic recurrence may be explained by our institution’s aggressive lymphadenectomy approach to EC, which was to perform complete lymphadenectomy exclusively and not performing lymph node sampling. In the present study, the median number of extracted lymph nodes was 58, the median number of the pelvic lymph nodes was 42.5, and 15.5 for the paraaortic region.

In patients with FIGO 1988 stage IIIA EC, we found that uterine serosal involvement was the only prognostic factor associated with recurrence. Serosal involvement increased the recurrence rate 5-fold. Slomovitz et al. (20) reported that non-endometrioid type and adnexal/serosal involvement was associated with diminished survival in their study in patients with FIGO 1988 stage IIIA EC. They found no effect of peritoneal cytology on survival (20). Similarly, Preyer et al. (12) reported that adnexal and serosal involvement in patients with stage IIIA EC according to FIGO 1988 was related with poor survival rates. However, they determined no effect of peritoneal cytology on survival.

The fundamental restriction of our study is its retrospective nature. Although the number of patients seems to be limited, it is an extensive number of patients when it comes to stage IIIA disease. In addition, this was a single-center study and the practice of complete lymphadenectomy maintains the homogeneity of the group.

In conclusion, recurrence is seen in one quarter of patients with stage IIIA EC according to FIGO 1988 criteria. Only uterine serosal involvement was related with adverse oncologic outcomes, not adnexal involvement or presence of positive cytology.

## Figures and Tables

**Table 1:**
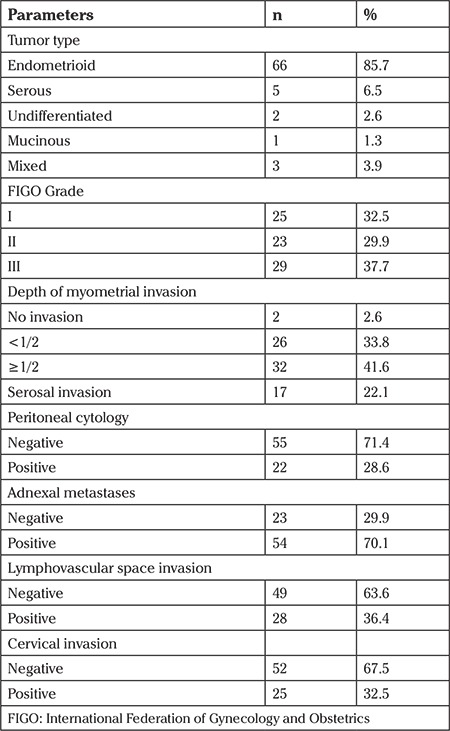
Pathologic characteristics of patients with stage IIIA disease according to the International Federation of Gynecology and Obstetrics 1988 staging system (n=77)

**Table 2:**
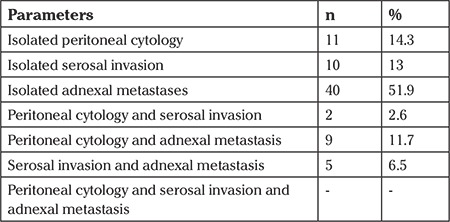
Pathologic features that necessitated the assignment of stage IIIA according to the International Federation of Gynecology and Obstetrics 1988 staging system

**Table 3:**
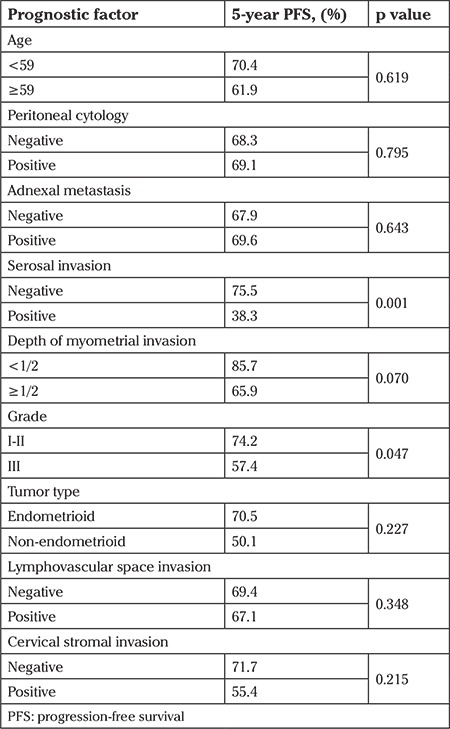
Univariate analysis comparing factors associated with progression-free survival

**Table 4:**
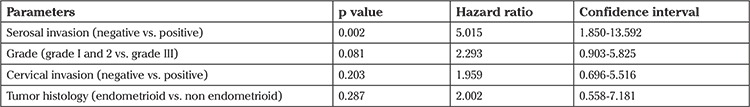
Multivariate analysis of factors determining recurrence
